# Cefiderocol Versus Best Available Therapy in the Treatment of Critically Ill Patients with Severe Infections Due to Resistant Gram-Negative Bacteria: A Systematic Review and Meta-Analysis

**DOI:** 10.3390/antibiotics13111048

**Published:** 2024-11-05

**Authors:** Carlos Risco-Risco, César Henriquez-Camacho, Marta Herrera-Rueda, José Barberán, David Andaluz-Ojeda

**Affiliations:** 1 Internal Medicine Department, University Hospital HM Sanchinarro, HM Hospitales, 28015 Madrid, Spain; 2 Internal Medicine Department, Móstoles University Hospital, 28935 Madrid, Spain; cesaraugusto.henriquez@salud.madrid.org (C.H.-C.);; 3 Faculty of Medicine, Francisco de Vitoria University, 28223 Madrid, Spain; 4 Internal Medicine Department, University Hospital HM Monteprincipe, HM Hospitales, Boadilla del Monte, 28660 Madrid, Spain; 5 HM Faculty of Health Sciences, Camilo Jose Cela University, 28692 Madrid, Spain; 6 Intensive Care Department, Palencia University Hospital Complex, 34004 Palencia, Spain; 7 Critical Care Area, University Hospital HM Sanchinarro, HM Hospitales, 28015 Madrid, Spain; 8 Group of Biomedical Research on Critical Care (BioCritic), Valladolid University, 47003 Valladolid, Spain

**Keywords:** severe infections, cefiderocol, *Acinetobacter baumannii*, critically ill, ICU, MDR, Gram-negative bacteria

## Abstract

Background: This study aims to assess the effectiveness and safety of cefiderocol in treating severe infections caused by multidrug-resistant Gram-negative bacteria (MDR-GNB) in critically ill patients, particularly those in intensive care units (ICUs). Methods: A meta-analysis of studies, including randomized clinical trials and observational studies in adult patients, was performed. Studies with at least 50% of critically ill patients were included. Studies with small sample size or without comparison groups were excluded. Sources included PubMed, Scopus, or Google Scholar, up to 14 August 2024. Risk of bias was assessed according to the Cochrane tool. The main outcome examined was 30-day mortality, while secondary outcomes assessed included clinical cure rates and adverse effects. Results were expressed with odds ratios. No funding was received for this study. It was registered in the International Prospective Register of Systematic Reviews (PROSPERO) with reference CRD42024563041. Results: eight studies, with 1339 patients were included in the meta-analysis. Cefiderocol treatment was associated with a lower 30-day mortality rate than other available therapies (pooled OR 0.47; 95% CI: 0.23–0.97, *p* = 0.04), particularly in cases of carbapenem-resistant *A. baumannii* infections (pooled OR 0.29; 95% CI: 0.14–0.60, *p* < 0.001). Although there was a non-significant trend toward higher clinical cure rates in the cefiderocol group (OR 1.59; 95% CI: 0.96–2.62, *p* = 0.07), the drug demonstrated at least non-inferiority when compared to other treatment options. Study limitations included moderate heterogeneity between studies, and a high risk of bias in non-RCT studies. (Five cohort studies were included). Another limitation is that five of the eight studies compared cefiderocol versus colistine, an antibiotic with known toxicity. Conclusions: The findings suggest that cefiderocol is a promising therapeutic option for managing severe MDR-GNB infections in critically ill patients, offering a potential global benefit on mortality and at least non-inferiority in the cure rate when compared with other therapies.

## 1. Introduction

The rise of multidrug-resistant (MDR) Gram-negative bacteria (GNB) stands as a significant global health challenge in the 21st century, making the treatment of infections caused by these microorganisms more difficult [1]. These microorganisms are difficult to treat because of their ability to develop resistance to multiple classes of antibiotics [2,3]. Non-fermenting Gram-negative bacteria, such as *Acinetobacter baumannii* and *Pseudomonas aeruginosa*, and carbapenem-resistant *Enterobacteriaceae* (CRE) are among the most concerning MDR pathogens due to their association with elevated rates of morbidity and mortality among hospitalized patients, especially those in intensive care unit (ICU) settings [4]. Among these pathogens, *Acinetobacter* has emerged as a particularly formidable foe because of its resistance to multiple antibiotics, including carbapenems. Recent research has revealed that carbapenem-resistant *A. baumannii* (CRAB) rates reach or exceed 50% in 21 countries across Southern and Eastern Europe [5]. When these microorganisms cause infections in more severe patients, such as those in the ICU setting, the problem is severely exacerbated because limited therapeutic options lead to high morbidity and mortality rates. Cefiderocol, a siderophore cephalosporin, has garnered attention because of its unique mechanism of action and potent activity against a wide range of MDR-GNB, including carbapenem-resistant strains [6].

Cefiderocol operates via a unique mechanism that distinguishes it from the other antibiotics. As a siderophore cephalosporin, in addition to passive diffusion through porin-mediated channels, it exploits the bacterial iron uptake system to penetrate bacterial cells, which allows high concentrations of the drug to be delivered to the site of cephalosporin action [2]. This mechanism not only enhances its uptake but also circumvents many traditional resistance mechanisms (porin deficiency, upregulation of efflux pump expression, and production of β-lactamases, including carbapenemases) employed by Gram-negative bacteria that limit the efficacy of other antibiotics. Its spectrum of activity encompasses a variety of problematic pathogens, MDR-GNB, such as *Pseudomonas aeruginosa, Acinetobacter baumannii, Stenotrophomonas maltophilia, and Enterobacterales*, which are commonly associated with nosocomial infections in ICU settings.

Several studies have demonstrated the clinical efficacy of cefiderocol against a variety of MDR-GNB [7,8,9]. However, most pivotal trials of cefiderocol have focused on patients with moderate or non-severe infections. Most studies on cefiderocol in ICU settings are limited to case series and a few retrospective cohort studies, making it difficult to draw robust conclusions regarding drug outcomes in this scenario [4,10]. Critically ill patients present different characteristics than other patients, which can alter the pharmacokinetics/pharmacodynamics (pK/pD) of many antibiotics. Situations, such as immunosuppression, increased volume of distribution, hypoalbuminemia, renal failure, and organ support measures, including continuous renal replacement therapy and extracorporeal membrane oxygenation, are frequently used in critically ill patients and may lead to both interpatient and intrapatient variabilities in drug pharmacokinetics, which can significantly alter antibiotic concentrations, thereby affecting their effectiveness [11,12]. This highlights the need for a further investigation into cefiderocol efficacy in the ICU setting. This meta-analysis aimed to systematically review and analyze the existing literature on the effectiveness and safety of cefiderocol in treating severe infections, paying special attention to critically ill patients. By consolidating data from various studies, we seek to provide a robust assessment of the role of cefiderocol in managing these challenging infections, thereby aiding clinicians in making evidence-based decisions.

## 2. Results

**Figure 1:**
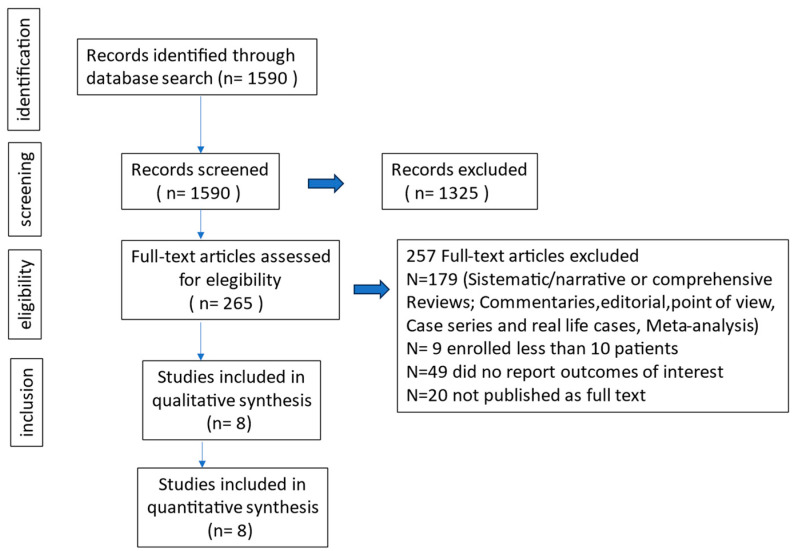
PRISMA flow diagram of the process of identification and selection of articles included in the meta-analysis.

We screened 1590 original works, of which 1325 articles were excluded after reviewing the titles and abstracts; 265 papers were obtained for full-text review. Following the exclusion of 257 papers that did not meet the inclusion criteria, eight studies were ultimately included in the analysis [13,14,15,16,17,18,19,20]. The process of article selection is illustrated in Figure 1.

Three studies were multicenter RCTs [13,14,15], four were single-center retrospective cohort studies [16,17,19,20], and the remaining study corresponded to a single-center prospective cohort [18]. The sample sizes varied between 73 and 371 patients, amounting to a total of 1339 participants. Five of the studies were carried out in Italy [16,17,18,19,20], while the others were multinational, involving centers from the US, Europe, and Asia [13,14,15]. Lower respiratory tract infections were the most frequently reported, with five studies including cases of ventilator-associated pneumonia (VAP) or hospital-acquired pneumonia (HAP) [15,18,19,20]. One study included bloodstream infections [17], another included complicated UTI or acute pyelonephritis [13], and the rest of the studies included different types of severe infections in ICU settings. It is worth noting that most studies (5/8) included selectively severe CRAB infections, regardless of the infection site [16,17,18,19,20]. A total of 702 patients were treated with cefiderocol, whereas 637 received other therapies. Cefiderocol was compared with colistin-containing regimens in four studies [16,17,18,19] all on severe CRAB infections. In two studies, cefiderocol was compared with carbapenems [13,15], and in the other two studies [14,20], with best available therapy (BAT).

With respect to the outcomes assessed, 30-day mortality was examined in seven studies, with the exception of one [13]. Table 1 and Table 2 present the description of the studies, together with the overall 30-day mortality rate for the entire population studied, encompassing all treatment protocols, while in four studies the clinical cure rate was compared [13,14,15,18].

### 2.1. Quality Appraisal of Studies Included in the Analysis

**Figure 2:**
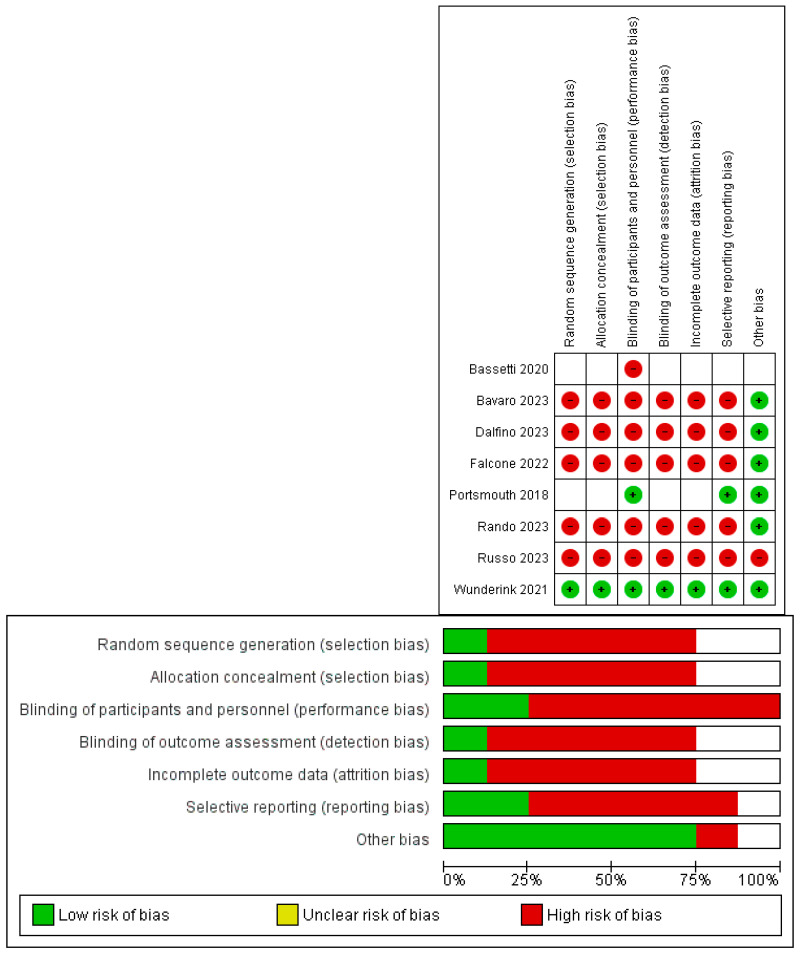
Risk of bias summary and bias graph.

The three RCTs that included all but one were judged to have a low risk of bias for at least three domains, while the remaining study (Basetti, 2020 only provided information in the text to evaluate one domain (performing bias). Five observational studies, including a comparator group, were judged to have a high risk of bias according to the Newcastle–Ottawa scale. Finally, among the eight studies, six were deemed to have a high risk of bias, one had an intermediate risk [13], and only Wunderink et al. [15] showed a low risk of bias. The quality assessment of the included studies is shown in Figure 2.

Publication bias was explored using a sensible test (Egger) combined with a specific test (Peters). Both tests showed no evidence for publication bias: Egger test *p* = 0.658, Peters test *p* = 0.385.

### 2.2. Meta-Analysis of Studies Evaluating Mortality

When we compared cefiderocol monotherapy versus other therapies among the seven studies that analyzed mortality [14,15,16,17,18,19,20], we observed a 28-day mortality rate of 31% among 702 patients receiving cefiderocol versus 48.2% among the 637 patients treated with other therapeutic regimens (*p* < 0.001). In the random effects model, the cumulative OR was 0.47(95% CI: 0.23–0.97, *p* = 0.04), in favor of cefiderocol.

**Figure 3:**
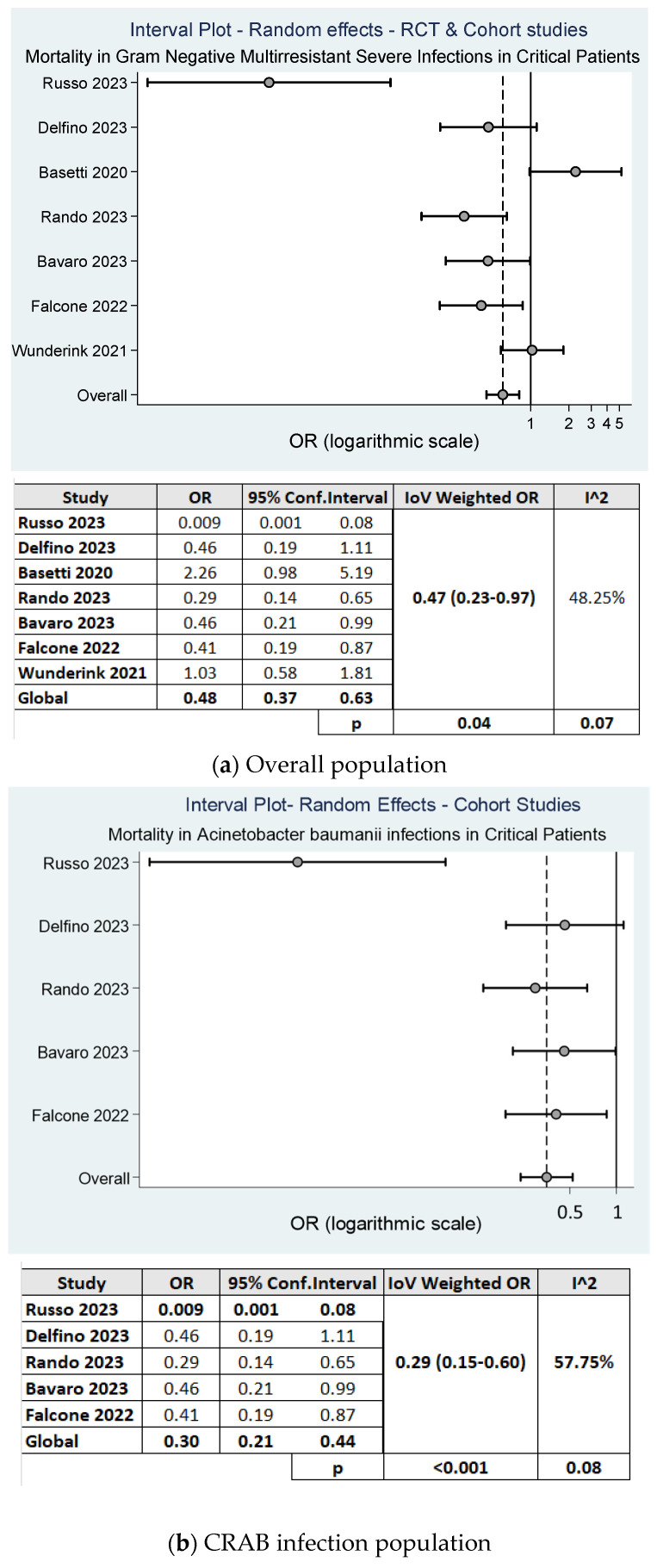

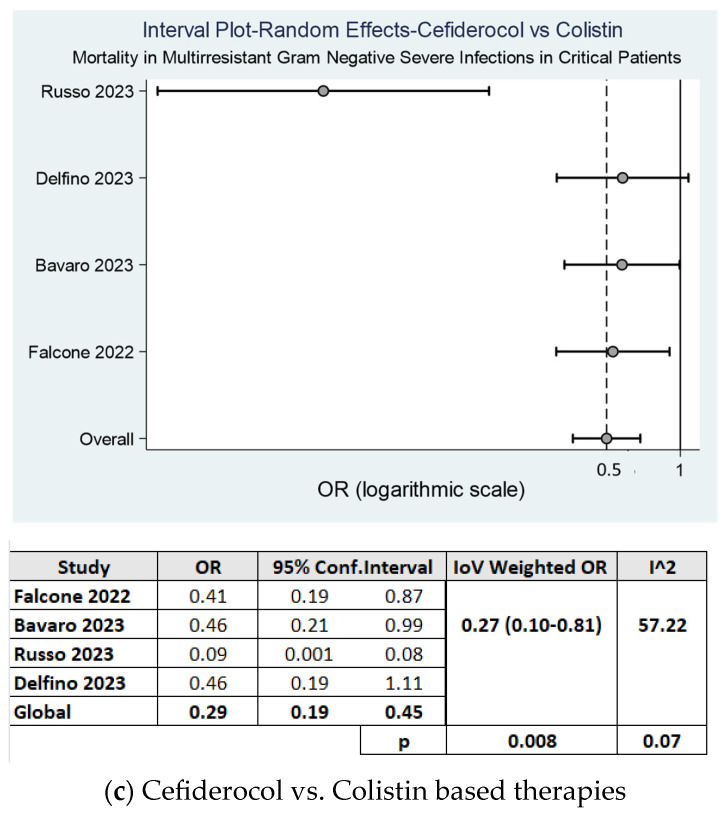
Meta-analysis of studies evaluating the 30-day mortality rate in patients treated with cefiderocol.

Moderate heterogeneity was detected between the studies (I^2^= 48.25%, *p* = 0.07). These findings were more robust in a sub-analysis of studies that enrolled only patients with CRAB infections [16,17,18,19,20]. In this scenario, we observed a significantly lower cumulative OR for a 30 day-mortality rate among the 204 patients receiving cefiderocol compared to 277 patients treated with BAT (OR 0.29; 95% CI: 0.14–0.60; *p* < 0.001) (Figure 3b). Moderate heterogeneity was observed between the groups (I^2^ = 51%, *p* = 0.08).

Four out of seven studies reported mortality using colistin as a comparator. We performed a sub-analysis comparing cefiderocol- and colistin-based therapies and found a cumulative OR for 30-day mortality of 0.27 (95% CI: 0.19–0.81, *p* = 0.008), with moderate heterogeneity between studies (I^2^ = 57%, *p* = 0.07) (Figure 3c).

### 2.3. Meta-Analysis of Studies Evaluating Microbiological Failure and Clinical Cure

**Figure 4:**
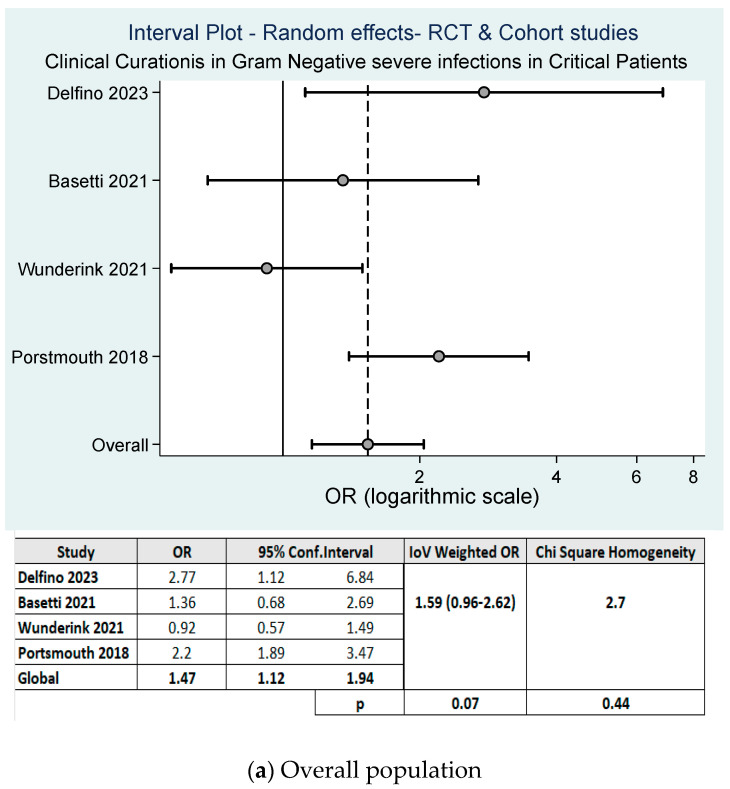

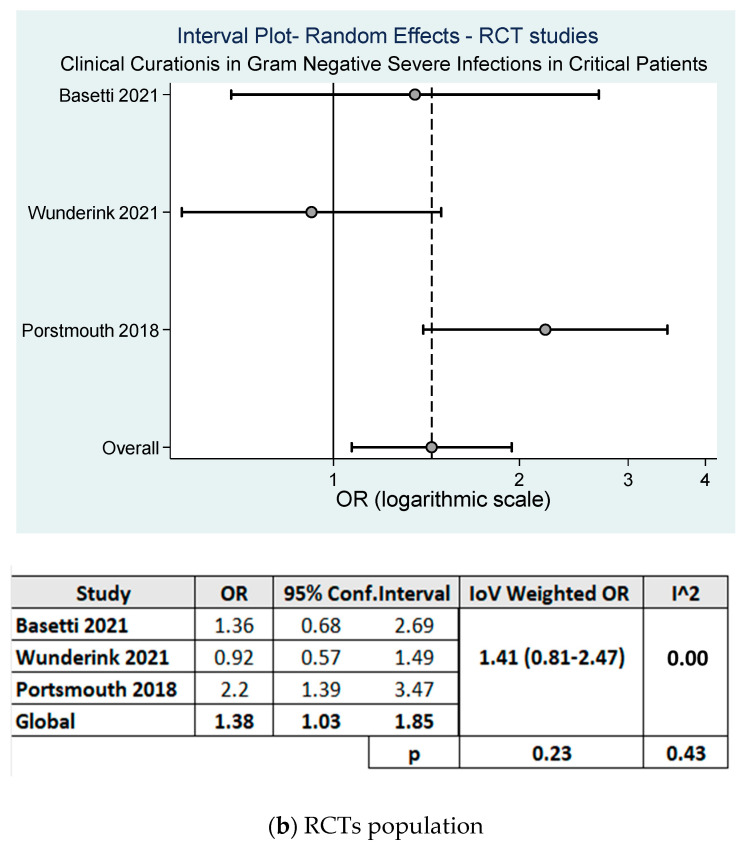
Meta-analysis of studies evaluating the curation rate in patients treated with cefiderocol vs. best available therapy.

Only four of the eight studies [13,14,15,18] included in this meta-analysis (three RCTs and one cohort study) described the composite of clinical and microbiological outcomes at the test of cure (i.e., 7 days after treatment cessation) as the primary or secondary endpoint, which was used to establish the non-inferiority of cefiderocol versus the other available therapies. Considering these four studies globally, the meta-analysis of the cure rate showed a percentage of cure in the cefiderocol group of 66.91% versus 57.81% in the BAT group (*p* = 0.005). On the random effects model, the cumulative OR of cure was 1.59 (95% CI: 0.96–2.62, *p*-value = 0.07), in favor of cefiderocol with low heterogeneity between the studies [I^2^ = 2.77%, *p* = 0.44] (Figure 4a).

The superiority of cefiderocol was not evidenced in the sub-analysis, which included only patients enrolled in the three RCTs (lower risk of bias). In this case, the cumulative OR was 1.41 (95% CI: 0.81–2.47, *p*-value = 0.23) without showing significant differences in the rates of cure between both groups, and with low heterogeneity between the studies (I^2^ = 0.0, *p* = 0.43) (Figure 4b).

## 3. Discussion

The findings from this meta-analysis indicate that patients receiving cefiderocol for severe MDR-GNB infections experience more favorable clinical outcomes compared to those treated with best available therapy (BAT). Our results show a notably reduced 30-day mortality rate in the cefiderocol group and a non-significant trend towards a higher clinical cure rate in this group, with at least non-inferiority compared to the other treatments. Among the eight studies included in this systematic review, only three were RCTs, and five were observational studies. The risk of bias was considered high in all studies except for two of the three RCTs [13,14].

The overall mortality results of our meta-analysis were favorable for cefiderocol. We found a significantly favorable effect, with greater survival in the cefiderocol group, regardless of the pathogen type or site of infection. The lower mortality in the cefiderocol group was consistent both in the overall population and in studies that included only severe CRAB infections. However, neither of the two RCTs [14,15] that evaluated 30-day mortality showed a benefit in favor of cefiderocol. The CREDIBLE-CR study [15] was a phase 3, multicenter trial focused on the efficacy and safety of cefiderocol for treating severe carbapenem-resistant Gram-negative infections. It demonstrated that cefiderocol had clinical and microbiological effectiveness comparable to that of best available therapy (BAT). However, in cases involving *Acinetobacter* spp. infections, the study found a higher overall mortality rate with cefiderocol compared to BAT (49% vs. 18%, respectively), particularly among patients with pneumonia, bloodstream infections, or severe sepsis due to CRAB. This disparity might be attributed to an uneven distribution of patients, as those on cefiderocol had a higher incidence of septic shock (26% vs. 6% in the BAT group) and longer ICU stays at the time of randomization (81% vs. 47%), indicating a greater baseline risk for mortality. Another randomized controlled trial, the APEKS-NP study [14], was a multicenter, double-blind, parallel-group phase 3 trial involving adult patients with hospital-acquired or ventilator-associated Gram-negative pneumonia. Participants were randomly assigned to receive either 3-hour intravenous infusions of cefiderocol (2 g) or meropenem (2 g) every 8 h for 7–14 days. This study concluded that cefiderocol was non-inferior to meropenem, with mortality rates of 12.4% and 11.6%, respectively.

Thus, the overall mortality results in favor of cefiderocol in our meta-analysis can be attributed to the remaining studies included. Although these were all observational studies, most of them had a retrospective design, and all found a lower mortality rate in the cefiderocol group than in the control group. It is important to emphasize that most of these studies were conducted entirely in ICU settings [16,18,19,20] and mainly included patients with a diagnosis of VAP due to CRAB. However, none of the RCTs analyzed in this meta-analysis exclusively included critically ill patients. In fact, Bassetti et al. [14] and Wunderink et al. [15] included 52% and 68% of critically ill patients at randomization, respectively, which likely suggests a lower or overall disease severity in these studies and therefore a better prognosis than those included in the aforementioned observational studies. Another circumstance that may influence our results is that in most of the observational studies included in this meta-analysis, the control arm used therapeutic regimens based on colistin as BAT. This drug has known harmful side effects and an unfavorable PK/PD profile, which could impact clinical outcomes. Nevertheless, a significant body of recent preclinical data from in vitro and in vivo studies indicates a potential clinical benefit of polymyxin-based treatments, either as monotherapy or in combination. These therapies have demonstrated synergistic activity against extensively drug-resistant (XDR) and difficult-to-treat resistant (DTR) strains of *P. aeruginosa, Klebsiella pneumoniae, and A. baumannii,* supporting their use in such cases. [21,22].

Regarding clinical cure, our meta-analysis showed a trend towards a higher cure rate (not statistically significant) in the cefiderocol group; however, this trend was not observed when considering only the RCTs, although cefiderocol still demonstrated non-inferiority in this outcome compared to BAT. Only four of the eight studies [13,14,15,18] included in the meta-analysis reported cure rates, which significantly limits the observation of this finding. Of the four aforementioned studies, two (Basetti et al. and Wunderink et al.) [14,15] did not find any difference in the cure rate in favor of cefiderocol. The previously mentioned limitations in these two trial studies, particularly those in the CREDIBLE-CR study [15], may also significantly influence the findings at this endpoint. Recent in vitro studies and real-world case series have shown a high rate of microbiological eradication and clinical cure when using cefiderocol to treat MDR-GNB in the ICU setting. In a recent study by Fendian et al., involving a cohort of critically ill patients with severe infections caused by MDR- or XDR-GNB, clinical cure was achieved in 90% of patients, while microbiological cure was seen in 80% [4]. Other studies have reported similar results [10,23]. Recent narrative reviews and expert commentaries have analyzed preclinical data, case series, and specific real-life scenarios in the ICU setting, such as continuous renal replacement therapy, ECMO, and immunosuppression, suggesting a benefit of cefiderocol over other treatments [24,25,26,27,28,29].

Notably, the findings of this meta-analysis align with the data recently reported by the authors of the PERSEUS study [30]. This retrospective, multicenter observational study was designed to evaluate the efficacy and safety of cefiderocol in real-world settings for adult patients with GNB infections. It included 261 critically ill adult patients with limited treatment options, all of whom received cefiderocol for ≥72 h for confirmed GNB infections. Of these patients, 64.8% were resistant to all tested antibiotics, and 44.4% had failed prior antibiotic treatments. The cohort was severely ill, with 63.2% in the ICU, 47.1% on mechanical ventilation, and 28% suffering from septic shock. The study found that patients treated with cefiderocol achieved an overall clinical success rate (defined as a combination of clinical cure and/or survival at day 28) of 84.3%, with a 28-day all-cause mortality rate of 21.5% [30]. The majority of patients had respiratory tract infections (47.9%) primarily caused by pathogens such as *Pseudomonas aeruginosa* (66.7%), *Klebsiella pneumoniae* (10.0%), and *Stenotrophomonas maltophilia* (7.7%).

These findings, including those concerning mortality and clinical cure, are in line with the results of our study. As far as we know, no other meta-analysis has specifically explored the role of cefiderocol in critically ill patients with MDR-GNB infections. Hsueh et al. [31] conducted a meta-analysis that included one phase 2 and two phase 3 trials, with the primary outcome focused on clinical response at the test of cure. Cefiderocol achieved clinical and microbiological responses comparable to those of the comparator agents. However, this work did not specifically focus on infections caused by MDR-GNB or on critically ill patients. In two recent meta-analyses, Onorato et al. [32] and Gatti et al. [8] analyzed cefiderocol-based regimens versus BAT in the treatment of CRAB infections. None of the studies focused on critically ill patients, but similar to our findings, they observed a significantly lower mortality rate [RR: 0.74 (95% CI: 0.57–0.95) and OR: 0.53 (95% CI: 0.39–0.71)] in the groups treated with cefiderocol compared to BAT. No differences were observed in the rates of microbiological and clinical failure.

Despite this, our meta-analysis has several limitations that should prompt cautious interpretation of our findings. Few high-quality studies have analyzed the role of cefiderocol in critically ill patients. Limited evidence is available on cohort studies with a high risk of bias. Many of these studies are limited to CRAB infections, and only a few address infections caused by other MDR-GNB, such as *Enterobacteriaceae* or other non-fermenting GNB. Additionally, in many cases, the comparator is colistin, and no studies have compared cefiderocol with newer antibiotics, including beta-lactamase inhibitors.

Under these circumstances, our conclusions should not be generalized because of the lack of additional trials to address these gaps. It is also necessary to investigate this drug further in a specific population of critically ill patients. However, based on our findings and recent evidence, cefiderocol has proven to be safe and effective, presenting a promising and viable option for treating MDR Gram-negative infections in the ICU setting.

## 4. Material and Methods

### 4.1. Search Strategy and Selection Criteria

We performed a systematic review and meta-analysis, including randomized controlled trials (RCTs) and observational cohort studies, comparing the mortality rate and other clinical outcomes (such as clinical cure rate) among patients with severe infections, preferably in ICU settings due to MDR-GNB, receiving cefiderocol or best available therapy. Our study adhered to the PRISMA guidelines [33] and was registered in the International Prospective Register of Systematic Reviews (PROSPERO) under the reference CRD42024563041.

Two authors (C.R.-R. and D.A.O.) conducted a search of original reports published up to August 2024 using MEDLINE, Scopus, and Google Scholar. The term ‘cefiderocol’ was used to identify studies for inclusion in the analysis. The following search strategy was used:

PubMed (last access 14 August 2024): (“Cefiderocol” AND “ICU”) OR (“Cefiderocol” AND “Critical Care”)) OR (“Cefiderocol” AND “Severe Infection”) OR (“Cefiderocol” AND “critically ill”) OR (“Cefiderocol” AND “nosocomial pneumonia”) OR (“Cefiderocol” AND “ventilator associated pneumonia”) OR (“Ceftiderocol” AND “sepsis”) OR (“Cefiderocol” AND “bloodstream infection”)

Google Scholar (last access 14 August 2024): “Cefiderocol” AND (“ICU” OR “Critical Care” OR “Severe Infection”) AND (“RCT “ OR “Cohort”)

Scopus (last access 14 August 2024): “Cefiderocol” AND “Severe Infections” “Cefiderocol” AND “Critical Care”, “Cefiderocol” AND ”ICU”, “Cefiderocol” AND “Critically ill patients”

The results of this search were assessed by two independent reviewers to choose the studies ultimately included in the systematic review and meta-analysis.

Studies referenced in the bibliographies of all relevant papers from the initial search were also considered for inclusion. Two researchers (C.R.-R. and D.A.O.) independently reviewed the titles and abstracts of all citations to select the articles for analysis, documenting the reasons for excluding each study. Thereafter, they retrieved articles previously selected to evaluate them for inclusion as full-text articles. In cases of disagreement, a third author (CJHC) made a definite decision.

### 4.2. Inclusion and Exclusion Criteria

Randomized controlled trials (RCTs) or observational cohort studies were selected if they included adult patients with severe infections caused by MDR-GNB admitted to the ICU, comparing treatment with cefiderocol versus other therapeutic alternatives. The selected studies must also provide data on mortality and/or clinical cure. Given the absence of RCTs exclusively involving critically ill patients, we decided to include those in which at least 50% of the study population were critically ill, with the remainder presenting severe infections meeting sepsis criteria.

Studies were excluded under the following conditions: (i) studies where less than 50% of the participants were critically ill; (ii) studies with fewer than 10 participants; (iii) studies lacking a comparison group; (iv) observational studies without multivariate analysis adjusting for potential confounders; (v) studies not published as full papers; (vi) meta-analyses, letters, reviews, meeting abstracts, or editorial comments; and (vii) studies not published in English.

### 4.3. Data Analysis

The quality of all studies was evaluated using established criteria, and the risk of bias for each study was assessed with RevMan tool, version 5.4.1, following Cochrane guidelines [34]. (CHC performed this assessment). We employed random effects models to estimate the combined effect from aggregate data due to heterogeneity between studies [35].

Most of the studies provided estimates of the risk of death or clinical cure. (ORs or 28–30 days HRs). We pooled these data based on raw data on mortality or clinical cure in different studies and obtained combined results with adjusted ORs (with 95% confidence intervals). Graphical representations of results were performed with the interval plot method. All data used in the meta-analyses were extracted from the published data included in the final selected articles.

Observational studies and RCTs were presented in separate interval plots categorized by study design. Heterogeneity between studies was evaluated using I^2^ statistics, which indicate the percentage of variation across studies attributable to heterogeneity rather than random chance [36]. In order to overcome possible bias due to heterogeneity between groups, subgroup meta-analysis was performed: only RCT studies, only studies addressing *Acinetobacter baumanii*, or only studies with colistin treatment as the control group. Publication bias was assessed using a combination of a sensible test (Egger) and a specific test (Peters). Statistical analysis was performed using the software STATA v. 16.2.

## 5. Conclusions

The emergence of MDR-GNB represents a significant challenge in the management of severe infections in critically ill patients. Cefiderocol, with its unique mechanism of action, has demonstrated efficacy and is a promising option for treating these infections. Although the risk of bias was considered high, our data seem to strengthen the efficacy profile of cefiderocol in severe MDR-GNB infections, especially those occurring in ICU settings, with a global benefit on mortality and at least non-inferiority in the cure rate when compared with other available therapies. However, more randomized clinical trials focusing exclusively on critically ill patients are needed to compare the action of this drug with the other best available therapies for the treatment of this type of infection to better allocate this novel drug in the real-world practice.

## Figures and Tables

**Table 1:** **General Information of Studies and Populations.** RCT: Randomized clinical trials, BAT: Best available therapy, PI: Principal investigator, ICU: Intensive care unit, HAP: Hospital acquired pneumonia; HCAP: Health care-associated pneumonia, VAP: Ventilator associated pneumonia CRAB: Carbapenem resistant *Acinetobacter baumannii*; UTI: Urinary tract infection; BSI: Blood stream infections.

Reference	Settings	Study Period (Follow-Up)	Study Design	N. Patients	Median Age (Years)	Male (%)
Portsmouth 2018 [13]	International (PI: USA)	Feb 2015–Aug 2016 (14 days)	RCT multicenter (67 centers) double blind	371	Cefiderocol:62.3 Imipenem-Cilastatin: 61.3	Cefiderocol:47 Imipenem-Cilastatin:48
Basetti 2020 [14]	International (PI: Italy)	Sep 2016–Apr 2019 (14 days)	RCT multicenter (95 centres) open label	150	Cefiderocol:63.1 BAT:63	Cefiderocol:65 BAT:29
Wunderink 2021 [15]	International (PI: USA)	Oct 2017–Apr 2019 (14–21 days)	RCT multicenter (17 centers) double blind	291	Cefiderocol:64.6 Meropenem:65.4	Cefiderocol:68 Meropenem:69
Falcone 2022 [16]	Italy	Jan 2020–Aug 2021 (30 days)	Single-center retrospective cohort	124	Cefiderocol:63 Colistin:68	Cefiderocol:61.7 Colistin:81.8
Bavaro 2023 [17]	Italy	Jan 2020–Dec 2022 (90 days)	Single-center retrospective cohort	118	Cefiderocol:69 Colistin:71	Cefiderocol:60 Colistin:59
Dalfino 2023 [18]	Italy	Mar 2021–Dec 2022 (28 days)	Single-center prospective	90	Cefiderocol:67 Non-Cefiderocol: 64	Cefiderocol:86 Colistin:38
Russo 2023 [19]	Italy	Mar 2020–Aug 2022 (30 days)	Single-center retrospective cohort adjusted by propensity score	73	Cefidercol:67 Colistin:60	Cefiderocol:26.3 Colistin regimen:24
Rando 2023 [20]	Italy	Sep 2020–Dec 2022 (28 days)	Single-center retrospective cohort	121	Cefiderocol:64 BAT:68	Cefiderocol: 76 BAT:77

**Table 2:** **Treatments, Type of Infection, and Clinical Outcomes.** BAT: Best available therapy, PI: Principal investigator, ICU: Intensive care unit, HAP: Hospital acquired pneumonia; HCAP: Health care-associated pneumonia, VAP: Ventilator associated pneumonia CRAB: Carbapenem resistant *Acinetobacter baumannii*; UTI: Urinary tract infection; BSI: Blood stream infection.

Reference	Type of Infection	Treatments	Mortality (%)	Cure Rate (%)
Portsmouth 2018 [13]	Complicated UTI or acute pyelonephritis	Cefiderocol vs. Imipenem-Cilastatin	Not reported	Cefiderocol:72.6% Imipenem: 54.6%
Basetti 2020 [14]	HAP, VAP, HCAP, BSI, or complicated UTI (carbapenem-resistant GNB)	Cefiderocol vs. Best Available Therapy (BAT)	Cefiderocol: 34% BAT: 18%	Cefiderocol:52.5%, BAT: 44.9%
Wunderink 2021 [15]	VAP, HAP, or HCAP (Gram-negative)	Cefiderocol vs. Meropenem	Cefiderocol: 21%, Meropenem: 20%	Cefiderocol: 64.8% Meropenem: 66.6%
Falcone 2022 [17]	Severe CRAB infections	Cefiderocol vs. Colistin	Cefiderocol: 34%, Colistin: 55%	Not reported
Bavaro 2023 [17]	BSI caused by CRAB	Cefiderocol vs. Colistin	Cefiderocol: 40%, Colistin: 59%	Not reported
Dalfino 2023 [18]	VAP due to CRAB	Cefiderocol + inhaled colistin vs. Colistin	Cefiderocol: 35% Colistin: 52%	Cefiderocol: 75% Colistin: 52%
Russo 2023 [19]	VAP and BSI in COVID-19 ICU patients	Cefiderocol vs. Colistin	Cefiderocol: 32% Colistin: 98%	Not reported
Rando 2023 [20]	CRAB-VAP in critically ill COVID patients	Cefiderocol vs. BAT	Cefiderocol: 44% BAT: 67%	Not reported

## Data Availability

Raw data are available upon reasonable request from the corresponding authors.
